# 

**Published:** 1865-10

**Authors:** 


### CONTENTS.

### ORIGINAL CONTRIBUTIONS.

ART. XXXIII. Treatment of Stricture of the Urethra by Forcible Rupture, ---- 593
ART. XXXIV. Surgical Cases. By E. Andrews, M.D., Prof. of Surgery in Chicago Medical College, ---- 596
ART. XXXV. Continued Fever.—The Type of Disease now Prevalent.—Cases.—Injurious Effects of Evacuants in the Early Stage, &c.; Being the Abstract of a Clinic in the Medical Wards of the Mercy Hospital. By N. S. Davis, M.D., Prof. of Clinical Medicine, &c., Chicago, ---- 600
ART. XXXVI. Patenting of Surgical Instruments. By Z. ---- 608

### SELECTIONS.

On a New Remedy for Dysentery. By William Kerr, Surgeon, &c., &c., ---- 609
Cases of Cerebro-Spinal Meningitis in Post Hospital, Gallop's Island, Boston Harbor, from September, 1864, to May, 1865. By Calvin G. Page, M.D., ---- 615
The Food and the Teeth.—Observations on the Inorganic Constituents of the Food of Children, as connected with the Decay of the Teeth, and the Physical Constitution of Women in America. By the late James Paul, M.D., of Trenton, N.J., ---- 623
An Eastern Antidote against Fever, ---- 599

### EDITORIAL.

Medical Colleges, ---- 634
Medical Colleges in Cincinnati, ---- 635
Explanation, ---- 635
The Mortality Statistics for August, 1865, ---- 636
The Mortality Statistics for September, 1865, ---- 637

